# Optimization of Ferimzone and Tricyclazole Analysis in Rice Straw Using QuEChERS Method and Its Application in UAV-Sprayed Residue Study

**DOI:** 10.3390/foods13213517

**Published:** 2024-11-04

**Authors:** So-Hee Kim, Jae-Woon Baek, Hye-Ran Eun, Ye-Jin Lee, Su-Min Kim, Mun-Ju Jeong, Yoon-Hee Lee, Hyun Ho Noh, Yongho Shin

**Affiliations:** 1 Residual Agrochemical Assessment Division, National Institute of Agricultural Sciences, Wanju 55365, Republic of Korea; ksh8607@korea.kr; 2 Department of Applied Bioscience, Dong-A University, Busan 49315, Republic of Korea

**Keywords:** rice straw, unmanned aerial vehicle, ferimzone, tricyclazole, LC-MS/MS

## Abstract

Rice straw is used as livestock feed and compost. Ferimzone and tricyclazole, common fungicides for rice blast control, can be found in high concentrations in rice straw after unmanned aerial vehicle (UAV) spraying, potentially affecting livestock and human health through pesticide residues. In this study, an optimized method for the analysis of the two fungicides in rice straw was developed using the improved QuEChERS method. After the optimization of water and solvent volume, extraction conditions including ethyl acetate (EtOAc), acetonitrile (MeCN), a mixed solvent, and MeCN containing 1% acetic acid were compared. Different salts, including unbuffered sodium chloride, citrate, and acetate buffer salts, were compared for partitioning. Among the preparation methods, the MeCN/EtOAc mixture with unbuffered salts showed the highest recovery rates (88.1–97.9%, RSD ≤ 5.1%). To address the severe matrix effect (%ME) of rice straw, which is characterized by low moisture content and cellulose-based complex matrices, samples were purified using 25 mg each of primary–secondary amine (PSA) and octadecylsilane (C_18_), without pesticide loss. The developed method was validated with a limit of quantification (LOQ) of 0.005 mg/kg for target pesticides, and recovery rates at levels of 0.01, 0.1, and 2 mg/kg met the permissible range (82.3–98.9%, RSD ≤ 8.3%). The %ME ranged from −17.6% to −0.3%, indicating a negligible effect. This optimized method was subsequently applied to residue studies following multi-rotor spraying. Fungicides from all fields and treatment groups during harvest season did not exceed the maximum residue limits (MRLs) for livestock feed. This confirms that UAV spraying can be safely managed without causing excessive residues.

## 1. Introduction

Globally, rice is a vital crop, providing a fundamental source of nutrition for approximately 50% of the world’s population [1,2]. This is particularly true in Asian countries, including the Republic of Korea, where rice is a major dietary component [3]. Enhancing rice production is essential for both national economies and food security [4,5]. Over recent decades, advancements in agricultural technology have steadily increased rice productivity, helping to sustain the rapidly growing global population [6].

A byproduct of the rice harvest, rice straw, is not merely a waste material but an important resource [7,8]. Rice straw is utilized as livestock feed, allowing rice-producing farms to support both crop and livestock production [9]. Additionally, it serves as a soil amendment, promoting sustainable agricultural practices [10]. Beyond its agricultural uses, rice straw is employed in various industrial applications, contributing substantial economic value [11].

To enhance agricultural productivity and safeguard crops against pests, the use of pesticides is indispensable [12,13]. However, the presence of pesticide residues poses significant concerns for food safety and environmental protection [14,15]. Effective monitoring and management of these residues are crucial to safeguarding human health and the environment [16,17]. Rice straw with high levels of pesticide residues used as animal feed can adversely affect livestock health, which in turn may impact human health [18]. Consequently, it is essential to monitor pesticide residues not only in rice but also in straw [19].

The MAFRA of South Korea, through the ‘Control of Livestock and Fish Feed Act,’ has delineated maximum residue limits (MRLs) for various feeds [20]. Since 2014, pesticide MRLs specific to rice straw used as animal feed have been separately established and monitored [20]. The guidelines, outlined in ‘Pesticide MRLs in Rice Straw for Cattle Feed’, set MRLs for 39 different pesticides, including ferimzone and tricyclazole. They are prominent fungicides extensively employed in rice cultivation. Ferimzone belongs to the pyrimidine class, while tricyclazole is categorized under the triazolobenzothiazole class. Ferimzone’s active ingredient primarily exists in the *Z* form, with its photolysis transformation product, the *E* isomer, also being present [21]. Both pesticides are effectively used to control rice diseases, including blast disease and leaf spot disease [22,23] The absence of established methods for the specific analysis of ferimzone and tricyclazole in rice straw poses a significant challenge in assessing and managing the safety of these pesticides [24].

Rice straw is characterized by its low moisture content and complex matrix resulting from the drying process, thus it presents significant analytical challenges. Specifically, the lower the moisture content relative to the total mass of the sample, the more pronounced the matrix effect becomes [25]. When employing mass spectrometry coupled with chromatography, there is a substantial risk of interference effects, such as ion suppression or enhancement. To mitigate these issues, it is crucial to eliminate as much of the matrix as possible during the sample cleanup process. QuEChERS (quick, easy, cheap, effective, rugged, and safe) is a sample preparation method widely utilized across various fields of research. Introduced by Anastassiades et al. (2003) [26], it offers simplified procedures and requires fewer materials compared to traditional sample preparation techniques, thereby enhancing time and cost efficiency. The purification process in the QuEChERS method should incorporate solid phase extraction (SPE) and dispersive-SPE (d-SPE) to remove the interferences in the samples and reduce matrix effects. SPE is renowned for its high selectivity and efficiency; however, it can be challenging to optimize the washing and elution conditions, and it is often time consuming [27]. To circumvent these limitations, conditioning-free SPE and filtration-type SPE have been developed [28]. On the other hand, d-SPE was introduced as a simpler, faster, and more cost-effective alternative to SPE. Despite its benefits, d-SPE may exhibit lower selectivity compared to SPE and may be less effective in purifying highly complex matrix samples. Therefore, it is crucial to select an appropriate d-SPE sorbent tailored to the specific sample or target pesticides.

Recent trends indicate a growing adoption of unmanned aerial vehicles (UAVs) for pesticide application in large-scale paddy fields, driven by the objectives of reducing labor costs and enhancing spray efficiency [29]. By eliminating the need for human entry into the fields, UAVs mitigate crop damage and lower the risk of pesticide exposure [30]. Additionally, UAVs can cover extensive areas faster than traditional pesticide application methods [31]. However, differences in pesticide residue levels may arise between aerial and manual applications due to factors such as the reduced dilution ratios (increased concentration) of the pesticide solution necessitated by the limited payload capacity of UAVs. Consequently, it is crucial to manage residue levels separately for each application method.

In this study, an analytical method was developed and validated for the accurate analysis of ferimzone and tricyclazole in rice straw. Liquid chromatography–tandem mass spectrometry (LC-MS/MS) was used to establish robust trace residue analysis techniques. Various extraction, partitioning, and cleanup conditions were evaluated during the QuEChERS sample preparation process to significantly enhance recovery rates and reduce matrix effects. The validated methods were subsequently applied to investigate residue levels after pesticide application using UAVs.

## 2. Materials and Methods

### 2.1. Chemicals and Reagents

Analytical grades of ferimzone *Z* and tricyclazole reference standards were sourced from Merck (Darmstadt, Germany) and Sigma-Aldrich (St. Louis, MO, USA), respectively. A ferimzone *E* stock solution (1000 mg/L) was obtained from AccuStandard Inc. (New Haven, CT, USA). LC-MS-grade distilled water was purchased from Merck (Darmstadt, Germany). The commercial pesticide product (agrochemical) was Nonsarang [ferimzone 15% + tricyclazole 8% suspension concentrate (SC); Kyung Nong, Seoul, Republic of Korea]. HPLC-grade ethyl acetate (EtOAc) and acetonitrile (MeCN) were acquired from Duksan Pure Chemical (Seoul, Republic of Korea). Acetic acid (HOAc, 99.5%) was obtained from Junsei Chemical Co., Ltd. (Tokyo, Japan). Methanol (MeOH; LC-MS grade) and formic acid (LC-MS grade) were obtained from Thermo Fisher Scientific (Waltham, MA, USA).

QuEChERS extraction kits were procured from Agilent Technologies (Santa Clara, CA, USA), including the Original method package [4 g of magnesium sulfate (MgSO_4_) and 1 g of sodium chloride (NaCl)], the citrate buffer package [EN-15662 method, 4 g of MgSO_4_, 1 g of NaCl, 1 g of sodium citrate (Na_3_Citrate·2H_2_O), and 0.5 g of sodium hydrogen citrate sesquihydrate (Na_2_HCitr·1.5H_2_O)], and the acetate buffer package [AOAC 2007.01 method, 6 g of MgSO_4_, and 1.5 g of sodium acetate (NaOAc)]. The Oasis PRiME hydrophilic–lipophilic balance (HLB) cartridge plus light (100 mg) was purchased from Waters Corporation (Milford, MA, USA). The d-SPE tubes including the primary–secondary amine (PSA) kit (25 mg of PSA and 150 mg of MgSO_4_), the octadecylsilane (C_18_) kit (25 mg of C_18_ and 150 mg of MgSO_4_), the PSA + C_18_ kit (25 mg of PSA, 25 mg of C_18_, and 150 mg of MgSO_4_; 50 mg of PSA, 50 mg of C_18_, and 150 mg of MgSO_4_), and the PSA + graphitized carbon black (GCB) kit (25 mg of PSA, 2.5 mg of GCB, and 150 mg of MgSO_4_) were obtained from Agilent Technologies (Santa Clara, CA, USA).

### 2.2. Preparation of Standard Solutions and Matrix-Matched Standards

Each standard (STD) was dissolved in MeCN to prepare stock solutions at a concentration of 1000 mg/L. The commercially obtained ferimzone *E* stock solution was then combined with the prepared solutions to create a working solution at a concentration of 200 mg/L. The solution was then serially diluted to achieve concentrations of 10, 1, 0.5, 0.25, 0.1, 0.05, 0.025, 0.01, 0.005, and 0.0025 mg/L. To prepare the matrix-matched standards (MMSTDs), 0.3 mL of each working solution was mixed with 0.3 mL of control rice straw matrix extracts to obtain concentrations of 0.25, 0.125, 0.05, 0.025, 0.0125, 0.005, 0.0025, 0.00125, and 0.001 mg/L. One microliter of each MMSTD was then injected into LC-MS/MS. The association between signal intensities and nominal concentrations on the chromatogram was verified, followed by plotting the calibration curve.

### 2.3. LC-MS/MS Analytical Conditions

A Shimadzu LCMS-8040 triple quadrupole mass spectrometer (Kyoto, Japan) was employed for the analysis, in conjunction with a Nexera liquid chromatograph (Shimadzu). The liquid chromatograph system comprised a column oven (CTO-20A), communications bus module (CBM-20A), autosampler (SIL-30AC), pump (LC-30AD), and degasser (DGU-20A5). Pesticide separation was achieved using a Cadenza CD-C_18_ column (2 × 150 mm, 3 μm; Imtakt Corp., Kyoto, Japan). The column oven temperature was maintained at 40 °C, with a flow rate of 0.2 mL/min and an injection volume of 1 μL. The mobile phase consisted of water containing 0.1% formic acid (A) and MeOH containing 0.1% formic acid (B). The gradient elution program for mobile phase B was as follows: started at 80% for 0.2 min, ramped to 92% over the next 3.3 min, rapidly raised to 98% within 0.1 min, held at 98% for 2.4 min, then decreased back to 80% over 0.1 min, and equilibrated for an additional 3.4 min.

Positive electrospray ionization (ESI+) and multiple reaction monitoring (MRM) methods were employed to analyze the target pesticides. Argon (purity 99.999%) was employed as the collision-induced dissociation (CID) gas. The desolvation line (DL) and heat block temperatures were maintained at 250 and 400 °C, respectively. The drying and nebulizing gas flow rates were set at 15 and 3 L/min, respectively. Data processing was conducted using Shimadzu LabSolutions software version 5.60 SP2.

To obtain MRM transition profiles, a full scan mode was employed to scan the mass-to-charge ratio (*m*/*z*) ranging from 150 to 800 at a rate of 410 u/s. For each pesticide, the precursor ion was selected from the scan spectrum and subjected to a product ion scan at varying collision energies (CEs) to confirm its fragmentation pattern. The selection of the quantifier and qualifier ions under the optimal CE was conducted based on their sensitivity and selectivity.

### 2.4. Optimization of Water and Solvent Volumes Before Extraction

A 3 g rice straw sample was moisturized with 6, 9, and 12 mL of water and left to stand for 15 min, after which 6, 9, and 12 mL of MeCN were added. The water saturation of the sample was confirmed, as well as the separation of the aqueous and organic solvent layers during the extraction and liquid partitioning process.

### 2.5. Comparison of Extraction and Partitioning Methods

To identify the optimal conditions for extraction–partitioning procedures, extraction methods were compared using MeCN, 1% HOAc in MeCN, EtOAc, and a 1:1 (*v*/*v*) mixture of solvents. Some of the solvents were further tested with the addition of 0.1% formic acid. The extracted samples were partitioned using three QuEChERS types of salts: the original unbuffered package, the EN-15662 citrate-buffered package, and AOAC 2007.01 acetate-buffered package. Recovery and matrix effect studies were conducted to evaluate the extraction–partitioning methods without the cleanup procedure.

### 2.6. Comparative Analysis of Various Cleanup Methods

After the selection of the optimal extraction–partitioning methods, the samples underwent a cleanup process using different sorbents. Several sorbent combinations and amounts were compared as follows: Method A applied filtration using the HLB plus light; Method B used the PSA (25 mg) kit; Method C employed the C_18_ (25 mg) kit; Method D used the PSA (25 mg) and C_18_ (25 mg) kits; Method E employed the PSA (50 mg) and C_18_ (50 mg) kits; and Method F used the PSA (25 mg) and GCB (2.5 mg) kits. The recovery rates and matrix effects for the analytes were evaluated.

### 2.7. Statistics

The recovery rates and matrix effect of the analytical methods were compared using ANOVA and Tukey’s post-hoc test to determine statistical significance, with a significance level set at *p* < 0.05. These statistical analyses were conducted using R software (version 4.3.2). Results are presented with corresponding letters to indicate statistical significance, with different letters denoting significant differences between groups.

### 2.8. Established Preparation Method

**Figure 1:**
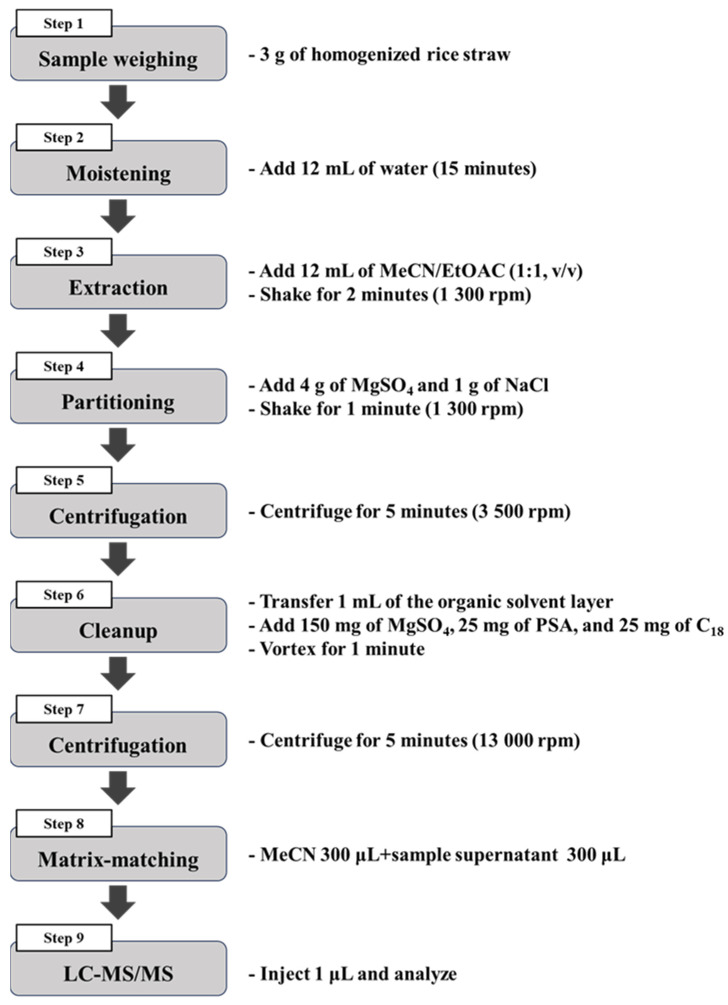
Diagram of the rice straw sample preparation process to analyze target pesticides.

Three grams of a homogenized rice straw sample were placed in a 50 mL centrifuge tube and saturated with 12 mL of water for 15 min. Subsequently, 12 mL of EtOAc/ MeCN (1:1, *v*/*v*) was added, and the mixture was shaken for 2 min at 1300 rpm using a Geno/Grinder (1600 Mini-G, SPEX SamplePrep, Metuchen, NJ, USA). Afterward, 1 g of NaCl and 4 g of MgSO_4_ were added, and the sample was shaken for 1 min at 1300 rpm. The mixture was then centrifuged at 3500 rpm for 5 min using a centrifuge (M15R, Hanil Scientific, Gimpo, Republic of Korea) to separate the liquid layers. An aliquot of 1 mL from the upper layer was transferred to a d-SPE tube containing 25 mg of PSA, 25 mg of C_18_, and 150 mg of MgSO_4_. This mixture was then agitated for 1 min and centrifuged at 13,000 rpm for 5 min using a microcentrifuge (1248, Labogene, Lillerød, Denmark). After allowing the solids to precipitate, 0.3 mL of the supernatant was combined with 0.3 mL of MeCN to compensate for matrix effects. Finally, 1 μL of the sample was injected into the LC-MS/MS system (Figure 1).

### 2.9. Method Validation

#### 2.9.1. Limit of Quantitation (LOQ) and Linearity of Calibration Curve

The LOQ was determined by analyzing MMSTD solutions at several concentrations, and the lowest concentration that satisfied the criteria for a signal-to-noise ratio (S/N) 10 or more was identified as the LOQ. To establish linearity, a series of MMSTD solutions were analyzed. The calibration curve was constructed by plotting the peak area or height versus the concentration of the analyte. The linearity was assessed by calculating the coefficient of determination (*r*^2^) of the calibration curve.

#### 2.9.2. Recovery and Storage Stability

A recovery validation experiment was performed at concentrations levels of 0.01 and 0.1 mg/kg. To achieve these concentrations in the samples, 0.03 mL of STDs at concentrations of 1 and 10 mg/L were spiked into 3 g of rice straw. The established method was applied for sample preparation, followed by matrix matching with MeCN (1:1, *v*:*v*). To evaluate the accuracy for the analytes exceeding the calibration curve range after dilution, 0.03 mL of a 200 mg/L working solution was spiked into 3 g of the sample to achieve a final concentration of 2 mg/kg. The 30 μL of extract solution obtained from the preparation was mixed with 270 μL of control sample extract and 0.3 mL of MeCN for matrix matching. Recovery rates were calculated based on the percentage ratio of the measured concentration to the theoretical concentration, with each sample analyzed in three repetitions (*n* = 3).

To evaluate the stability of target pesticides during sample storage, the control samples harvested from Buan-gun and Gunsan-Si (Jeonbuk-do, Republic of Korea) were spiked with STD solutions (0.1 mg/kg). These samples were stored for 43 days at −20 °C. This study was assessed for accuracy and precision using the methods described above.

#### 2.9.3. Matrix Effect

The matrix effect was calculated by substituting the slope of the calibration curve of the pure STD and the MMSTD into Equation (1) (%ME). A %ME value close to 0 indicates minimal matrix effects, whereas positive or negative values indicate signal enhancement or suppression, respectively. Matrix effects are categorized based on their %ME values. A soft matrix effect is defined by %ME within ±20%. Medium matrix effects are indicated by values ranging from −50% to −20% or from 20% to 50%. Values falling below −50% or exceeding 50% are classified as strong matrix effects.

(1) $$\%\;ME=\left(\frac{Slope\;of\;the\;matrix\;matched\;standard\;calibration\;curve}{Slope\;of\;the\;pure\;standard\;carlibration\;curve}-1\right)\times 100$$

### 2.10. Field Trial

Field trials were conducted in the paddy fields of Buan-gun (Field 1) and Gunsan-si (Field 2). The pesticide was applied using the YMR-08 multi-copter (YAMAHA, Shizuoka, Japan) equipped with XR-11002 nozzles (Teejet, Glendale Heights, IL, USA). The XR-11002 nozzle sprays with a droplet size of 263–340 µm, which reduces drift and provides optimal adhesion. It is the most commonly used nozzle in the Republic of Korea. The UAV operated at an altitude of 3 m, with an effective spraying width of 4 m and a speed of 11 km/h. The nozzle discharge rate was 160 mL/13 s at a pressure of 40 psi. Applications were conducted only when wind speeds were less than 2 m/s to ensure optimal conditions.

In accordance with the ‘safety usage standards’ [32], the pesticide was diluted 8-fold and applied three times before harvest. In addition to the standard procedures, three treatment plots were set up for comparison: Treatment group A received only the pesticide; group B was sprayed with a mixture of the pesticide and 1% Cares adjuvant (Dongbangagro, Seoul, Korea); group C was treated with the pesticide and 2% Gondor adjuvant (De Sangosse, Agen, France); and group D served as the untreated control group. Each plot was spaced 8 m apart, with three replications for each treatment. To ensure consistency and minimize temporal variation, three multi-copters were used simultaneously for the treatment groups.

Harvesting was conducted on different dates for the two fields: Field 1 was harvested on 12 October 2022, and Field 2 on 1 October 2022. The rice panicles and grains were separated from the straw, dried for 7 days at room temperature, homogenized with dry ice, and stored at −20 °C until analysis.

## 3. Results and Discussion

### 3.1. Optimization of MRM Transitions for LC-MS/MS

To optimize the MRM transitions for ferimzone isomers and tricyclazole, their precursor ions were initially identified using full scan analysis (Table 1). The monoisotopic mass of ferimzone is 254.2 g/mol, and that of tricyclazole is 189.0 g/mol. The spectra revealed that all of the pesticides were ionized as proton adduct forms ([M+H]^+^). Therefore, the precursor ion *m*/*z* values were determined to be 255.2 for ferimzone and 190.2 for tricyclazole. Using a product ion scan, the optimal product ions at varying CEs were evaluated. For ferimzone, the ion at *m*/*z* 132.2, which demonstrated the highest sensitivity, selectivity, and peak shape, was chosen as the quantifier, while the ion at *m*/*z* 91.1, being the second most suitable, was selected as the qualifier ion. Additional product ions were also detected at *m*/*z* 124.2 and 117.1. Tricyclazole was selected with *m*/*z* 163.1 as the quantifier ion and *m*/*z* 136.1 as the qualifier ion, with additional product ions detected at *m*/*z* 109.1 and 65.1.

**Figure 2:**
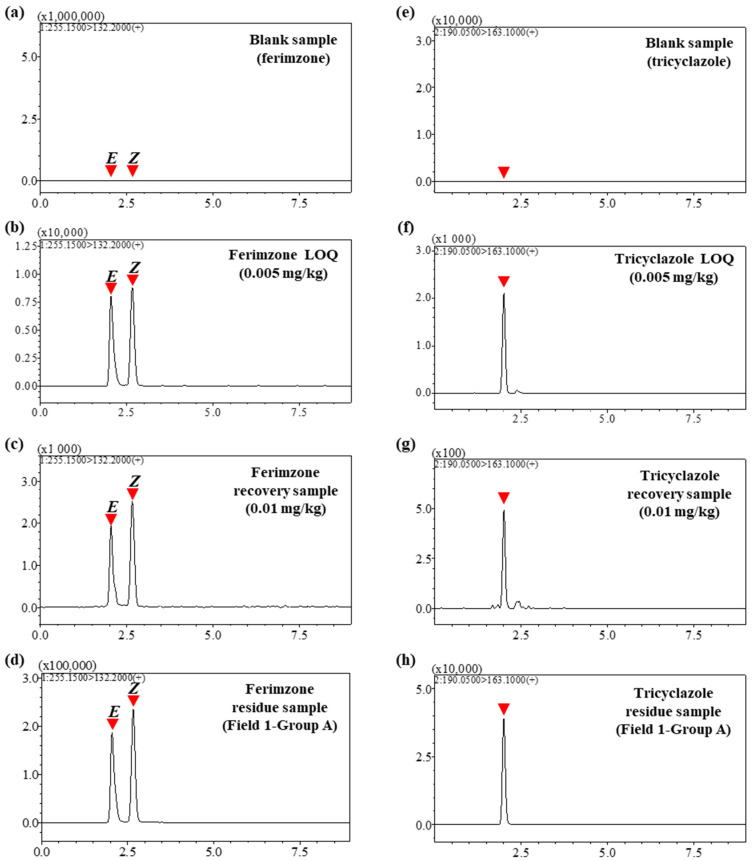
Chromatograms for ferimzone and tricyclazole in rice straw. Panels (**a**–**d**) display chromatograms for ferimzone, where the peak that eluted earlier corresponds to the *E* form and the later peak to the *Z* form. Panels (**e**–**h**) exhibit chromatograms for tricyclazole. Specifically, (**a**,**e**) depict blank samples without pesticide, (**b**,**f**) show matrix-matched standard (MMSTD) at the limit of quantification (LOQ, 0.005 mg/kg), (**c**,**g**) illustrate recovery samples at 0.01 mg/kg, and (**d**,**h**) represent residue samples from Group A in Field 1.

These results are consistent with previous studies utilizing LC-MS/MS [33,34]. Some studies have changed the quantitative and qualitative ions or selected additional product ions as the representative ions [35,36,37]. Such differences can arise due to the type of mass spectrometer, solvent composition, or CE values [38,39]. Using MRM transitions, chromatograms for each analyte were verified, with retention times ranging from 2.00 to 2.66 min. Notably, Ferimzone *Z* and *E*, which share the same MRM conditions, were completely separated to enable the quantification of individual compounds (Figure 2).

### 3.2. Sample Amount Selection and Moistening Water-Extraction Solvent Volume and Layer Separation

For dry or low water content materials such as grains, legumes, and soil, adequate water saturation prior to sample preparation is crucial to facilitate the extraction of pesticides [40]. Dried rice straw similarly requires a moistening process [19,41]. For the preparation of dried materials using QuEChERS, the recommended sample weight is typically 5 g [42]. In our preliminary experiments using 5 g of rice straw samples, 10 mL of water did not sufficiently saturate the sample. While the sample was adequately saturated with more than 15 mL of water, the addition of solvent and partitioning salts caused the sample mixture to fill the entire 50 mL centrifuge tube, making shaking impossible.

**Figure 3:**
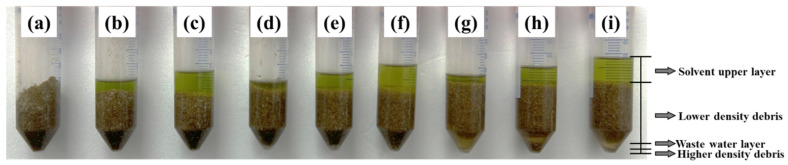
QuEChERS partitioning results based on the volume of water ((**a**–**c**) 6 mL, (**d**–**f**) 9 mL, (**g**–**i**) 12 mL) and organic solvent ((**a**,**d**,**g**) 6 mL; (**b**,**e**,**h**) 9 mL; (**c**,**f**,**i**) 12 mL) treated to the sample.

When 3 g of the sample was used, there was no excessive volume increase observed during preparation in any of the samples. The partitioning results of the samples after the addition of 6, 9, and 12 mL each of water and extraction solvent, followed by the addition of partitioning salts and centrifugation, are depicted in Figure 3. In the QuEChERS method, the optimal partitioning state is assessed based on the clear separation into distinct layers: the solvent layer, a low-density debris layer, the water layer, and a high-density debris layer [40]. Samples treated with 6 mL of water (Figure 3a–c) exhibited insufficient saturation, and the water layer was not observed. Samples moistened with 9 mL of water (d–f) were adequately saturated, but four-layer separation was not achieved. In contrast, samples treated with 12 mL of water (g–i) successfully separated into four distinct layers. Among these, samples treated with 3 mL of extraction solvent (g) were excluded due to the difficulty in collecting the supernatant layer. Ultimately, 12 mL of extraction solvent (i) was chosen, as it allows for easy collection of the supernatant and is expected to result in the least matrix effect.

### 3.3. Optimizing Extraction–Partitioning Methods

Depending on the characteristics of the pesticides and crop matrices, extraction solvents such as dichloromethane, hexane, acetone, EtOAc, MeCN, and MeOH are widely used [43,44,45,46]. Among them, EtOAc and MeCN are less harmful to human health, and they possess the appropriate polarity for reverse-phase LC systems. Under specific conditions, they can form separate layers with water, making it easier to eliminate polar interfering substances from the extracts [26]. Recently, a mixture of EtOAc and MeCN has been used to improve extraction efficiency in complex matrices such as herbal medicines [28].

The recovery rates of three pesticides were evaluated using various extraction solvents including MeCN, EtOAc, and their mixture (Table 2). When MeCN was employed as the solvent (Methods M1 and M2), the recovery rates ranged from 84.4% to 94.6%. For the EtOAc/MeCN mixed solvent (M3 and M4), recovery rates varied from 73.0% to 97.9%. Utilizing EtOAc alone (M5 and M6) resulted in recovery rates between 58.1% and 88.4%. The AOAC 2007.01 method (M7) produced recovery rates in the range of 83.1% to 88.1%. Notably, the recovery rates for tricyclazole using EtOAc alone were between 58.1% and 60.5%, failing to meet the acceptable recovery range of 70–120% as stipulated by the SANTE 11312/2021 guidelines [47]. The inclusion of citrate buffer in Methods M1–M4 did not enhance and often significantly decreased recovery rates, indicating that pH adjustment with buffers negatively impacted extraction efficiency. The highest recovery rates for all pesticides were achieved using the EtOAc/MeCN mixed solvent with unbuffered salts (M3), confirming its superior extraction efficiency for complex rice straw matrices.

By incorporating organic acid into the solvent mixture, we assessed the extraction efficiencies for the target analytes. This procedure is a well-established technique to enhance the recovery rates of acidic pesticides [48,49], as it suppresses the ionization of the analyte in the extracts. It is known that pesticides with lower pKa values exhibit higher extraction efficiencies in moderately acidified solvents [50]. Yuan et al. (2021) demonstrated that the recovery rate of tricyclazole in spinach was enhanced when formic acid was added to the MeCN extraction solvent [51]. Jeong et al. (2024) found that adding 0.1% formic acid to the EtOAc/MeCN mixture improved the extraction efficiency of ferimzone in rice samples [39].

**Figure 4:**
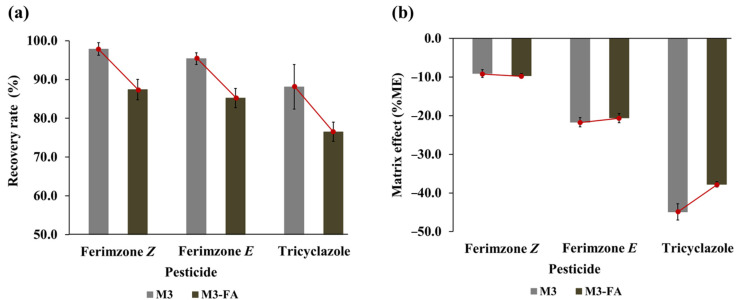
Comparison of recovery rates and matrix effect (%ME) for ferimzone isomers and tricyclazole using extraction solvents with and without 0.1% formic acid (FA). (**a**) Recovery rate. (**b**) Matrix effect. M3 refers the method shown in Table 2, while M3-FA denotes M3 with 0.1% FA in MeCN.

Contrary to these findings, our study observed a significant decrease in the recovery rates of all target pesticides upon the addition of 0.1% formic acid (Figure 4a). Xie et al. (2020) also noted a reduction in recovery rates when 0.1% formic acid was introduced to the MeCN extraction method for broflanilide in rice straw samples [52]. The complex matrices of rice straw may create a more challenging environment for the extraction solvents, leading to reduced efficiency. For instance, the presence of lignin and cellulose might cause stronger adsorption of pesticides, which could be exacerbated by the acidic conditions, possibly leading to stronger interactions between the pesticides and the straw matrices. Further investigation is needed to understand the underlying reasons for these discrepancies.

Although the addition of acid significantly increased the matrix effect (%ME) for tricyclazole (Figure 4b), it remained within the medium effect range. Therefore, implementing a cleanup procedure to mitigate the matrix effect would be more effective. Consequently, the method of treating formic acid was excluded from our procedure.

Despite the excellent recovery rates of pesticides using M3, significant signal suppression was still observed (Table S1). The matrix effect study results indicated that ferimzone *Z* exhibited a soft effect across all methods (−7.2% to −15.1%) in the crude extracts, whereas the ferimzone *E* and tricyclazole fell within the medium effect range (−21.4% to −49.0%). Notably, tricyclazole showed a matrix effect close approaching the strong effect level (−40.6% to −49.0%), suggesting a high likelihood of sample matrix influence during analysis. To mitigate the matrix effect to the target soft effect level, a cleanup process was deemed necessary.

### 3.4. Determination of the Optimal Cleanup Method

Rice straw contains a high concentration of compounds such as cellulose, hemicellulose, and lignin [53,54], which can induce a matrix effect [25]. If sample matrices are not adequately removed, impurities or macromolecules may accumulate and persist in the column, leading to potential issues. This can diminish measurement efficiency and, in severe cases, result in clogging the column. In this study, the crude extract from M3 was purified with six different cleanup methods (Table 3).

The recovery rates of ferimzone isomers across all cleanup methods ranged from 94.2% to 100.2%, indicating negligible loss during the cleanup process. In contrast, the recovery rate of tricyclazole fell below 80% for all methods except Method D. This indicates that tricyclazole was partially adsorbed onto the sorbents during cleanup. Specifically, a significant decrease in tricyclazole recovery was observed when the quantities of PSA and C_18_ were doubled (Method D vs. E). The decrease in recovery is likely due to the increased surface area and adsorption capacity of the sorbents, which enhances tricyclazole retention on the sorbent material. This finding aligns with the report by Georgakopoulos et al. (2011) and our previous study, which indicated that increasing the amount of sorbent during cleanup decreases the recovery rate [42,55]. Therefore, tricyclazole appears to be more susceptible to adsorption by PSA and C_18_, resulting in lower recovery rates at higher sorbent quantities.

The %ME values showed minimal change with the use of HLB compared to the sample before purification. When C_18_ alone was used as a sorbent, the matrix effect slightly reduced, although the %ME value for tricyclazole remained within the medium effect range at −35.9%. Methods B, D, and E, which involved the addition of PSA, exhibited %ME values within the soft effect range (−18.2% to 5.4%) for the target pesticides. However, the addition of GCB to PSA (Method F) resulted in a significant increase in %ME for tricyclazole compared to PSA alone. This increase is likely due to GCB’s unique adsorption properties, which may interact more strongly with certain matrix components, thereby elevating the overall matrix effect.

Based on the observed results, Method D was selected as the optimal cleanup method. This method provided high recovery rates for both ferimzone isomers and tricyclazole while maintaining acceptable %ME values. In the studies conducted by Im et al. (2015) and Lee et al. (2015) on the analysis of herbicides in rice straw, sample cleanup was performed using PSA and C_18_ combination methodologies [56,57].

### 3.5. Method Validation of the Established Method

Validation of the analytical method is crucial for applying a newly developed method to practical experiments. This process involves assessing sensitivity, reliability, accuracy/precision, and ruggedness using parameters such as LOQ, linearity of calibration curve, recovery rates, and matrix effects (Table 4).

#### 3.5.1. LOQ and Linearity

The minimum concentration ensuring S/N of 10 or more for ferimzone isomers and tricyclazole was determined to be 0.005 mg/kg (Figure 2b,f). The established method demonstrated superior sensitivity compared to previous studies for ferimzone (LOQ: 0.01 mg/kg for rice, wheat, barley, and oats; 0.066 mg/kg for soil) and tricyclazole (0.006 mg/kg for rice grain; 0.01 mg/kg for rice and rice flour) [58,59,60,61]. The MRL for ferimzone in rice straw for feed is 5 mg/kg, and for tricyclazole, 15 mg/kg. Given these MRLs, the method’s sensitivity ensures effective residue monitoring of target pesticides at levels well below these limits. The calibration range of the MMSTD curves was established between 0.005 and 1 mg/kg in the sample, with adjustments made using the 1/x weighting method. Linearity of the calibration curves was evaluated based on the coefficient of determination (*r*^2^). The *r*^2^ value quantifies the percentage of variation in the chromatogram signal that is attributable to changes in the analyte concentration [62]. As the variation in the signal due to concentration changes approaches 100%, the *r*^2^ value approaches 1. An *r*^2^ value close to 1 thus indicates a high correlation between concentration and signal. Ferimzone isomers and tricyclazole showed *r*^2^ values greater than 0.999, indicating a strong correlation (Table 4).

#### 3.5.2. Recovery and Storage Stability

Recovery studies were performed at concentration levels of 0.01, 0.1, and 2 mg/kg. The average recovery rates for target pesticides at each concentration ranged from 82.3% to 98.9% with RSD 0.8–5.2% (Table 4). The recovery rates satisfied the acceptable range of 70–120% (RSD ≤ 20%) as specified by the SANTE 11312/2021 guidelines [47]. These results demonstrate the method’s accuracy and consistency. Storage stability studies at −20 °C were conducted to assess pesticide degradation over a 43-day period (Table 4). The results were stable: ferimzone isomers showed a recovery rate of 106.5–114.0% (RSD 4.1–8.3%), and tricyclazole showed 80.2% (RSD 5.9%) This confirms that the pesticides remain stable under the tested storage conditions, ensuring reliable long-term analysis.

#### 3.5.3. Matrix Effect

The matrix effect evaluates the ruggedness of analytical methods. The %MEs observed in rice straw were −0.3% for ferimzone *Z*, −7.1% for ferimzone *E*, and −17.6% for tricyclazole (Table 4). All pesticides fell within the soft effect range (within ±20%), indicating that the matrix effect is negligible and ensuring the method’s ruggedness [63]. Previous reports indicated a %ME of −63% for broflanilide, 6.51–12.72% for flonicamid, and 3.88–12.09% for dinotefuran [64,65]. It is important to note that the matrix effects of the same pesticide on the same crop can vary among different cultivars [25,66]. Therefore, minimizing the presence of matrices that co-elute with the analytes is essential to prevent quantitative distortion.

### 3.6. Assessment of Pesticide Residue Levels in Rice Paddies Following UAV Spraying

The validated method was applied in a residue trial using UAVs to spray fungicides on rice paddies located in Buan-gun (Field 1) and Gunsan-si (Field 2), Republic of Korea. The residue levels for treatment group A, using the general UAV application method with agrochemical diluted 8-fold, were found to be 0.42–0.82 mg/kg for ferimzone and 0.20–0.66 mg/kg for tricyclazole (Table 5). Although the pesticide residue levels varied by approximately two to threefold between the two locations, they remained far below the MRL of 5–15 mg/kg. This confirms that the high-concentration UAV spraying method poses no safety concerns.

For groups B and C, the Cares and Gondor adjuvants were used, respectively. These adjuvants are designed to increase initial pesticide residue levels and reduce pesticide drift [42]. This rise in early residue levels raises concerns about the potential to exceed the MRL at harvest, making it essential to monitor during this period [67]. The results showed that for both groups, the residue levels ranged from 0.25–0.70 mg/kg for ferimzone and 0.10–0.66 mg/kg for tricyclazole. These levels were similar to or significantly lower than those of the untreated control group with no adjuvant. Taylor et al. (2019) reported that the addition of adjuvants to agrochemicals can either enhance or reduce the rate of volatilization [68]. The adjuvants used in this study likely contributed to increased volatilization of the pesticides, thereby maintaining residue levels within safe limits.

Previous studies have demonstrated that various adjuvants can influence pesticide residue levels in crops [40,69,70]. Nevertheless, there is a lack of comprehensive research identifying which specific factors in adjuvants most significantly impact crop residue levels and elucidating the mechanisms through which adjuvants affect pesticide dissipation [71,72]. Therefore, further investigation is required to understand why residue levels were lower in the groups treated with adjuvants compared to the group without treatment.

## 4. Conclusions

This study developed and validated an improved analytical method to enhance the efficiency of ferimzone isomers and tricyclazole analysis in rice straw. This method was then applied to pesticide residue studies using UAVs. In the sample preparation process, significant improvements in recovery results for target pesticides were achieved by extracting with a mixture of EtOAc/MeCN (1:1, *v*:*v*) and using unbuffered partitioning salts. For cleanup, d-SPE containing 25 mg each of PSA and C_18_ was selected due to its high effectiveness in reducing matrix effects without loss of analytes. The LOQ for ferimzone and tricyclazole in rice straw was determined to be 0.005 mg/kg. It demonstrated sufficient sensitivity to confirm whether residues exceeded these MRLs. Validation parameters, including recovery rate, storage stability, and matrix effects, met acceptable ranges, indicating the method’s reliability and ruggedness. This method was successfully applied to residue studies involving UAV-based pesticide application with/without adjuvants. Samples from all fields and groups did not exceed the regulated MRLs, demonstrating the method’s effectiveness in managing pesticide residue levels within safe levels. This research provides an optimal guideline for analysis of ferimzone and tricyclazole in rice straw, a matrix with low moisture content and complex composition. It also highlights the importance of managing pesticide residues in agricultural byproducts, which can affect livestock and human health.

## Figures and Tables

**Table 1:** MRM transitions and retention times (t_R_) for ferimzone isomers and tricyclazole.

Pesticide	t_R_ (min)	Monoisotopic Mass	Ionization Type of Precursor Ion	Precursor Ion > Product Ion (CE, V)	
				Quantifier	Qualifier
Ferimzone *Z*	2.66	254.2	[M+H]^+^	255.2 > 132.2 (20)	255.2 > 91.1 (31)
Ferimzone *E*	2.05	254.2	[M+H]^+^	255.2 > 132.2 (20)	255.2 > 91.1 (31)
Tricyclazole	2.00	189.0	[M+H]^+^	190.2 > 163.1 (21)	190.2 > 136.1 (27)

**Table 2:** Comparison of recovery rates at 0.1 mg/kg for target pesticides using different extraction solvents and partitioning salts.

Method	Extraction Solvent	Partitoning Salt	Recovery ± SD ^1^ (%, *n* = 3)		
			Ferimzone *Z*	Ferimzone *E*	Tricyclazole
M1	MeCN	Unbuffered ^3^	90.7 ± 2.0 bc ^6^	93.8 ± 1.7 ab	84.4 ± 0.7 a
M2	MeCN	Citrate buffer ^4^	94.6 ± 1.4 ab	82.4 ± 0.9 c	84.6 ± 3.9 a
M3	EtOAc/MeCN ^2^	Unbuffered	97.9 ± 1.6 a	95.4 ± 1.5 a	88.1 ± 5.1 a
M4	EtOAc/MeCN	Citrate buffer	94.0 ± 2.4 ab	87.1 ± 3.8 c	73.0 ± 1.8 b
M5	EtOAc	Unbuffered	87.6 ± 0.3 c	88.0 ± 2.8 bc	60.5 ± 1.0 c
M6	EtOAc	Citrate buffer	88.4 ± 1.9 c	87.3 ± 0.7 c	58.1 ± 1.7 c
M7	1% HOAc in MeCN	Acetate buffer ^5^	88.1 ± 0.6 c	83.3 ± 1.2 c	83.1 ± 2.2 a

^1^ Standard deviation, ^2^ Mixture of solvents (1:1, *v*/*v*), ^3^ Original method: 4 g of MgSO_4_ and 1 g of NaCl, ^4^ EN-15662 method: 4 g of MgSO_4_, 1 g of NaCl, 1 g of Na_3_Citr·2H_2_O, and 0.5 g of Na_2_HCitr·1.5H_2_O, ^5^ AOAC 2007.01 method: 6 g of MgSO_4_ and 1.5 g of NaOAc, ^6^ Significant differences (*p* < 0.05) are indicated by letters from a to c.

**Table 3:** Recovery rates and %MEs of ferimzone isomers and tricyclazole after cleanup with various d-SPE sorbents. The crude extracts were from the preparation using M3 methods.

Method	Cleanup Method	Recovery ± SD ^1^ (%, *n* = 3)			%ME ± SD (%, *n* = 3)		
		Ferimzone *Z*	Ferimzone *E*	Tricyclazole	Ferimzone *Z*	Ferimzone *E*	Tricyclazole
(A)	HLB ^2^	97.4 ± 1.7 ab ^8^	95.3 ± 1.2 b	79.3 ± 1.0 b	−12.3 ± 0.7 d	−22.6 ± 0.5 d	−42.2 ± 3.6 c
(B)	PSA (25 mg) ^3^	95.1 ± 0.6 ab	100.2 ± 0.7 a	78.0 ± 1.7 b	5.4 ± 1.5 a	−8.3 ± 1.1 ab	−16.3 ± 3.3 a
(C)	C_18_ (25 mg) ^4^	94.2 ± 0.3 b	96.1 ± 1.8 b	78.5 ± 1.6 b	−5.2 ± 1.6 c	−19.1 ± 0.9 c	−35.9 ± 1.4 c
(D)	PSA (25 mg) + C_18_ (25 mg) ^5^	99.7 ± 2.1 a	99.8 ± 0.5 a	84.3 ± 1.8 a	1.5 ± 1.8 ab	−8.4 ± 1.6 ab	−15.8 ± 1.0 a
(E)	PSA (50 mg) + C_18_ (50 mg) ^6^	98.3 ± 0.9 ab	100.1 ± 0.6 a	77.6 ± 2.2 b	1.0 ± 0.2 b	−7.0 ± 1.5 a	−18.2 ± 1.4 ab
(F)	PSA (25 mg) + GCB (2.5 mg) ^7^	96.4 ± 3.4 ab	98.5 ± 2.3 ab	79.1 ± 1.6 b	1.1 ± 2.5 b	−10.6 ± 0.9 b	−24.1 ± 2.1 b

^1^ Standard deviation, ^2^ PRiME hydrophilic–lipophilic balance cartridge plus light, ^3^ 25 mg of PSA and 150 mg of MgSO_4_, ^4^ 25 mg of C_18_ and 150 mg of MgSO_4_, ^5^ 25 mg of PSA, 25 mg of C_18_, and 150 mg of MgSO_4_, ^6^ 50 mg of PSA, 50 mg of C_18_, and 150 mg of MgSO_4_, ^7^ 25 mg of PSA, 2.5 mg of GCB, and 150 mg of MgSO_4_, ^8^ Significant differences (*p* < 0.05) are indicated by letters from a to d.

**Table 4:** Recovery and storage stability of ferimzone isomers and tricyclazole in rice straw.

Pesticide	LOQ (mg/kg)	Linearity (*r*^2^)	%ME	Accuracy and Precision Study	Treated Level (mg/kg)	Storage Period (Days)	Accuracy (%)	RSD ^1^ (*n* = 3) (%)
Ferimzone *Z*	0.005	0.9997	−0.3	Recovery	0.01	-	98.9	2.6
				Recovery	0.1	-	97.8	1.4
				Recovery	2	-	96.7	1.1
				Storage stability	0.1	43	114.0	8.3
Ferimzone *E*	0.005	0.9999	−7.1	Recovery	0.01	-	98.7	5.2
				Recovery	0.1	-	95.8	0.9
				Recovery	2	-	93.3	0.8
				Storage stability	0.1	43	106.5	4.1
Tricyclazole	0.005	0.9993	−17.6	Recovery	0.01	-	87.7	4.3
				Recovery	0.1	-	85.5	1.9
				Recovery	2	-	82.3	1.4
				Storage stability	0.1	43	80.2	5.9

^1^ Relative standard deviation.

**Table 5:** Residue levels of ferimzone and tricyclazole in rice straw following UAV application across various fields and adjuvants.

Pesticide	Field	Group	Adjuvant	Concentration (mg/kg)					MRL ^2^ (mg/kg)
				Trial 1	Trial 2	Trial 3	Max	Mean ± SD ^1^	
Ferimzone ^3^	1 (Buan)	A	-	0.79	0.77	0.91	0.91	0.82 ± 0.08 a ^4^	5
		B	Cares	0.34	0.34	0.43	0.43	0.37 ± 0.05 b	
		C	Gondor	0.56	0.79	0.74	0.79	0.70 ± 0.12 a	
	2 (Gunsan)	A	-	0.34	0.42	0.51	0.51	0.42 ± 0.09 a	
		B	Cares	0.40	0.48	0.31	0.48	0.40 ± 0.09 a	
		C	Gondor	0.26	0.19	0.29	0.29	0.25 ± 0.05 a	
Tricyclazole	1 (Buan)	A	-	0.58	0.68	0.73	0.73	0.66 ± 0.08 a	15
		B	Cares	0.31	0.35	0.34	0.35	0.33 ± 0.02 b	
		C	Gondor	0.52	0.77	0.68	0.77	0.66 ± 0.13 a	
	2 (Gunsan)	A	-	0.19	0.18	0.23	0.23	0.20 ± 0.03 a	
		B	Cares	0.11	0.16	0.14	0.16	0.14 ± 0.03 b	
		C	Gondor	0.10	0.09	0.11	0.11	0.10 ± 0.01 b	

^1^ Standard deviation, ^2^ MRLs in rice straw for cattle feed, ^3^ The sum of the residues of ferimzone *Z* and *E*, ^4^ Significant differences (*p* < 0.05) are indicated by letters from a to b.

## Data Availability

The original contributions presented in the study are included in the article/Supplementary Materials; further inquiries can be directed to the corresponding authors.

## Supplementary Materials

The following supporting information can be downloaded at: https://www.mdpi.com/article/10.3390/foods13213517/s1, Table S1: Matrix effects (%MEs) of ferimzone isomers and tricyclazole after cross-use of extraction solvent and partitioning salts.
