# Supplementary material for: Optimization of Ferimzone and Tricyclazole Analysis in Rice Straw Using QuEChERS Method and Its Application in UAV-Sprayed Residue Study
Source: Foods. 2024 Nov 4;13(21):3517. doi: 10.3390/foods13213517 (PMC11545821; doi:10.3390/foods13213517)
Supplement: Supplementary file 1 [file foods-13-03517-s001.zip › foods-3217459-supplementary.pdf]

Supplementary Information

Table S1. Matrix effects (%MEs) of ferimzone isomers and tricyclazole after cross-use of extraction solvent and partitioning salts

Method	Extraction solvent	Partitoning Salt	%ME ± SD [1] (%, n = 3)		
			Ferimzone *Z*	Ferimzone *E*	Tricyclazole
M1	MeCN	Unbuffered [3]	–7.2 ± 2.1	–21.4 ± 0.5	–41.0 ± 1.3
M2	MeCN	Citrate-buffer [4]	–7.3 ± 0.1	–26.2 ± 0.2	–42.4 ± 2.6
M3	EtOAc/MeCN [2]	Unbuffered	–9.1 ± 1.0	–21.7 ± 1.2	–44.9 ± 2.1
M4	EtOAc/MeCN	Citrate-buffer	–6.5 ± 3.4	–22.6 ± 4.2	–40.6 ± 3.6
M5	EtOAc	Unbuffered	–12.0 ± 3.5	–33.5 ± 0.9	–48.8 ± 0.6
M6	EtOAc	Citrate-buffer	–15.1 ± 2.0	–32.6 ± 1.4	–49.0 ± 1.1
M7	1% HOAc in MeCN	Acetate-buffer [5]	–10.4 ± 4.0	–24.5 ± 2.1	–40.8 ± 0.2

[1] Standard deviation, [2] Mixture of solvents (1:1, v/v), [3] Original method: 4 g of $MgSO_4$ and 1 g of NaCl, [4] EN-15662 method: 4 g of $MgSO_4$, 1 g of NaCl, 1 g of $Na_3Citr{\cdot}2H_2O$, and 0.5 g of $Na_2HCitr{\cdot}1.5H_2O$, [5] AOAC 2007.01 method: 6 g of $MgSO_4$ and 1.5 g of NaOAc.
